# Amplitude cancellation influences the association between frequency components in the neural drive to muscle and the rectified EMG signal

**DOI:** 10.1371/journal.pcbi.1006985

**Published:** 2019-05-03

**Authors:** Jakob Lund Dideriksen, Dario Farina

**Affiliations:** 1 Department of Health Science and Technology, Aalborg University, Aalborg, Denmark; 2 Department of Bioengineering, Imperial College London, London, United Kingdom; Johns Hopkins University, UNITED STATES

## Abstract

The rectified surface EMG signal is commonly used as an estimator of the neural drive to muscles and therefore to infer sources of synaptic input to motor neurons. Loss of EMG amplitude due to the overlap of motor unit action potentials (amplitude cancellation), however, may distort the spectrum of the rectified EMG and thereby its correlation with the neural drive. In this study, we investigated the impact of amplitude cancelation on this correlation using analytical derivations and a computational model of motor neuron activity, force, and the EMG signal. First, we demonstrated analytically that an ideal rectified EMG signal without amplitude cancellation (EMG_nc_) is superior to the actual rectified EMG signal as estimator of the neural drive to muscle. This observation was confirmed by the simulations, as the average coefficient of determination (r^2^) between the neural drive in the 1–30 Hz band and EMG_nc_ (0.59±0.08) was matched by the correlation between the rectified EMG and the neural drive only when the level of amplitude cancellation was low (<40%) at low contraction levels (<5% of maximum voluntary contraction force; MVC). This correlation, however, decreased linearly with amplitude cancellation (r = -0.83) to values of r^2^ <0.2 at amplitude cancellation levels >60% (contraction levels >15% MVC). Moreover, the simulations showed that a stronger (i.e. more variable) neural drive implied a stronger correlation between the rectified EMG and the neural drive and that amplitude cancellation distorted this correlation mainly for low-frequency components (<5 Hz) of the neural drive. In conclusion, the results indicate that amplitude cancellation distorts the spectrum of the rectified EMG signal. This implies that valid use of the rectified EMG as an estimator of the neural drive requires low contraction levels and/or strong common synaptic input to the motor neurons.

## Author summary

The rectified surface EMG signal is commonly used to analyze the neural activation of muscles. However, since this signal is most often exposed to so-called amplitude cancellation (loss of EMG amplitude due to overlap of positive and negative phases of different motor unit action potentials), the frequency content of the rectified EMG may not fully reflect that of the neural drive to the muscle. In this study we prove this notion analytically and demonstrate, using simulations, that the rectified EMG signal accurately reflects the neural drive to the muscle only in a limited set of conditions. Specifically, these conditions include low contraction levels and/or high variability of the neural drive. In other conditions, the rectified EMG signal from a muscle is a poor predictor of its neural input. This finding has potentially large implications for the way neural drive to muscles and neural connectivity (e.g. across muscles or between the brain and a muscle) should be analyzed.

## Introduction

The pool of motor neurons innervating a muscle receives a large relative proportion of common synaptic input [1]. The common input is transmitted to the motor neuron output generating the neural drive to the muscle, which is the ensemble of discharge timings of the motor neurons of the pool. The input-output transmission of common input is approximately linear in many conditions [2–4]. Consequently, the analysis of the neural drive to muscles by motor unit recordings has the potential to reveal neural connectivity, e.g. between pools of motor neurons innervating different muscles or between motor neurons and the motor cortex [5,6]. Moreover, the low-frequency components of the neural drive accurately predicts the fluctuations in the isometric force produced by the muscle [7]. Although the neural drive to a muscle can be measured directly from motor unit recordings [8–10], it is more often indirectly inferred from surface electromyographic (EMG) signals.

The amplitude of the EMG signal is associated to the strength of the neural drive. However, it is also influenced by the waveforms of the motor unit action potentials that depend on anatomy and electrode positioning, among other factors [11,12]. The influence of the shape of the action potentials can be partly reduced by standardizing the recording configurations and normalizing the EMG signal. However, the motor unit action potentials also act as high-pass filters of the neural drive (with a cut-off frequency in the range 15–35 Hz) and therefore may reduce the power of low-frequency components of the neural drive [13]. For this reason, rectification of the EMG signal is typically recommended [14,15]. While rectification may recover low frequencies, it introduces distortion in the power spectrum of the EMG signal due to amplitude cancellation [13]. Amplitude cancellation can be defined as the difference in amplitude between the sum of the rectified motor unit action potential trains (no-cancellation condition, which cannot be experimentally measured) and the rectified EMG (that can be actually measured) and can therefore be modeled as a signal-dependent noise term influencing the spectral properties of the rectified EMG [16]. Simulation studies have shown that amplitude cancellation implies a reduction of >50% in the EMG amplitude (i.e., the standard deviation of the EMG or square root of signal power) with respect to the no-cancellation EMG, even at low contraction levels [17,18]. Since the difference between no-cancellation EMG and rectified EMG has a colored spectrum [13], amplitude cancellation may limit the possibility of accurately inferring individual frequency components of the neural drive to muscle from the rectified EMG [13,18]. The degree of this distortion in the rectified EMG power spectrum is however not yet understood since amplitude cancellation has been previously investigated in terms of its total power but not of the distribution of its power across frequency bands.

**Fig 1:**
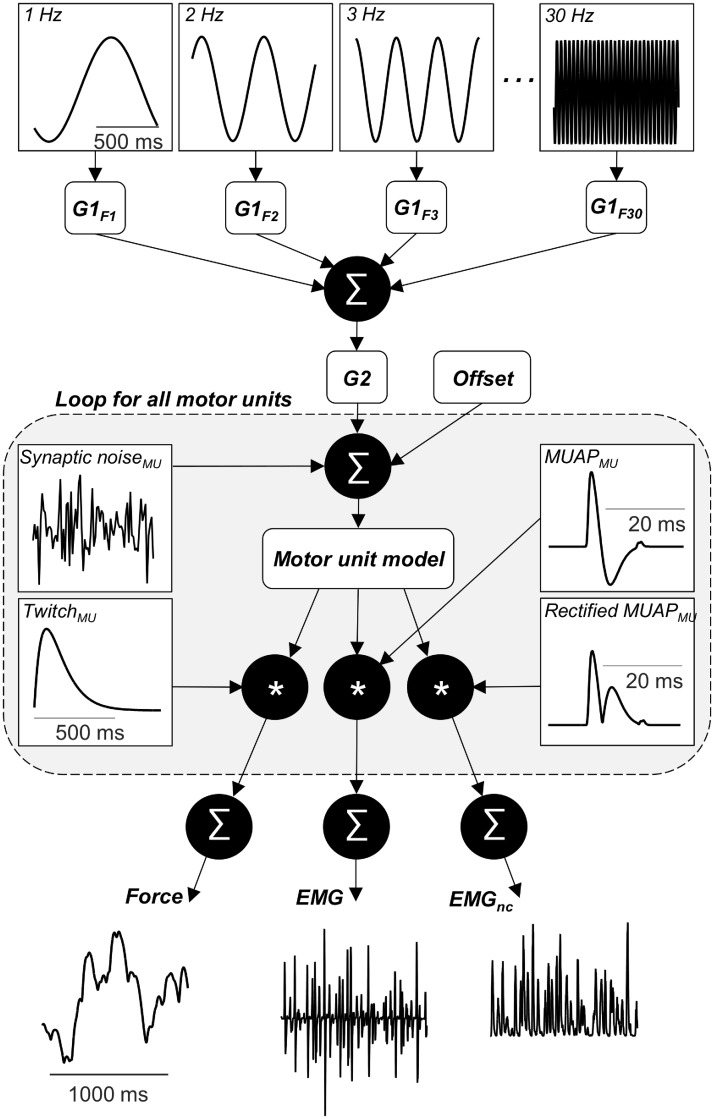
In the model, the common synaptic input to the motor neurons is the sum of 30 sine waves (frequencies: 1–30 Hz), each with an amplitude determined by a gain (G1_F1-F30_) that is determined by the values of a random variable with uniform distribution between 0 and 1. The gain G2 determined the average power of the common input (low, medium, or high) and an offset was added that determined the average contraction level. For each motor unit, independent synaptic noise was added before the motor unit model determined the motor unit spike trains (timing of motor neuron action potentials). For each motor unit, the spike train was convolved with pre-defined templates for the motor unit twitch forces, and the raw and rectified motor unit action potentials. By summation of the force and the EMG signals for each motor unit, the force, EMG, and EMG_nc_ for the full muscle were obtained. The cumulative spike train (CST) was calculated as the algebraic sum of all motor unit spike trains (neural drive to the muscle).

Here, we present a theoretical and simulation study that unravels the effect of amplitude cancellation on the frequency components of the rectified EMG. The simulations were based on a realistic computational model that characterized a motor neuron population and the surface EMG and force generated by the muscle it innervates (Fig 1). Two surface EMG signals were simulated. The regular EMG as well as a no-cancellation EMG (EMG_nc_) obtained by rectification of action potentials prior to summation across motor units. Importantly, this implies that EMG_nc_ cannot be measured experimentally, but can only be obtained in simulations. The correlations between the simulated neural drive to the muscle (the ensemble of discharge timings of all motor neurons) and the two simulated EMG signals were quantified to reveal the degree to which this relation was affected by amplitude cancellation. Together, the theoretical and the simulation analyses demonstrate that amplitude cancellation in many conditions disrupts the ability of the rectified surface EMG signal to reflect the neural drive in an accurate way.

## Results

### Theoretical analysis

We can approximate the rectified EMG (|EMG|) signal as:

|EMG(t)|=|s(t)*p(t)| (1)

and the no-cancellation EMG (EMG_nc_) as:

$$EMG_{nc}(t)=s(t)*\left|p(t)\right|,$$ (2)

where *t* denotes time, *s(t)* is the neural drive to the muscle, mathematically represented as a series of delta functions centered at the discharge instants of the motor neurons, and *p(t)* is the average action potential across all motor units. These approximations correspond to assuming that all motor units have the same action potential, as the average waveform among all action potential shapes. The effect of amplitude cancellation can be characterized by the signal c*(t)*, which we define as the difference between the rectified and the no-cancellation EMG:

$$c(t)=EMG_{nc}(t)-\left|EMG(t)\right|=s(t)*\left|p(t)\right|-\left|s(t)*p(t)\right|$$ (3)

Amplitude cancellation is the amplitude of the signal *c(t)*.

First, we consider the cross-correlation function (*R(τ)*) between the no-cancellation EMG and the neural drive to the muscle. This can be derived as follows [19]:

$$\begin{array}{l} R_{EMG_{nc},s}(\tau )=E\{EMG_{nc}(t)\cdot s(t+\tau )\}\\ =E\{s(t)*\left|p(t)\right|\cdot s(t+\tau )\}\\ =E\{\int_{-\infty }^{\infty }s(\alpha )\cdot \left|p(t-\alpha )\right|d\alpha \cdot s(t+\tau )\}\\ =E\{\int_{-\infty }^{\infty }s(\alpha )\cdot s(t+\tau )\cdot \left|p(t-\alpha )\right|d\alpha \}\\ =\int_{-\infty }^{\infty }R_{ss}(t-\alpha +\tau )\cdot \left|p(t-\alpha )\right|d\alpha \end{array}$$ (4)

By the substitution *β = t-α*, Eq 4 can be re-written as:

$$\begin{array}{l} R_{EMG_{nc},s}(\tau )=\int_{-\infty }^{\infty }R_{ss}(\beta +\tau )\cdot \left|p(\beta )\right|d\beta \\ =R_{ss}(-\tau )*\left|p(\tau )\right| \end{array}$$ (5)

Since *R*_*ss*_*(τ)* is symmetric, Eq 5 can be re-written as:

$$R_{EMG_{nc},s}(\tau )=R_{ss}(\tau )*\left|p(\tau )\right|$$ (6)

The cross spectrum (*G*) between *EMG*_*n*c_ and *s(t)* is the Fourier transform of the cross-correlation function [19]:

$$\begin{array}{l} G_{EMG_{nc},s}(f)=F\{R_{EMG_{nc},s}(\tau )\}\\ =F\{R_{ss}(\tau )*\left|p(\tau )\right|\}\\ =G_{ss}(f)\cdot H_{P}(f), \end{array}$$ (7)

where *F{·}* is the Fourier transform operator, *G*_*ss*_*(f)* is the cross spectrum of the neural drive and *H*_*p*_*(f)* is the power spectrum of the rectified action potential. Eq 7 implies that the cross spectrum between *EMG*_*nc*_ and *s(t)* is equivalent to *G*_*ss*_ only if *H*_*p*_*(f)* = 1 for all *f*. In this case, the coherence between the no-cancellation EMG and the neural drive to muscle (cross-spectrum normalized by auto-spectra) would be equal to 1 for all frequencies. This is the case only if the rectified action potential is a delta function, thus not altering the neural drive. Therefore, a perfect correlation between *EMG*_*nc*_ and the neural drive never occurs. Instead, the level of correlation depends on the spectrum of the action potential.

Next, we consider the correlation between the rectified EMG and the neural drive. This is derived as follows:

$$\begin{array}{l} R_{\left|EMG\right|,s}(\tau )=E\{\left|EMG(t)\right|\cdot s(t+\tau )\}\\ =E\{\left|s(t)*p(t)\right|\cdot s(t+\tau )\}\\ =E\{\left|\int_{-\infty }^{\infty }p(\alpha )\cdot s(t-\alpha )d\alpha \right|\cdot s(t+\tau )\} \end{array}$$ (8)

Since *s(t)* ≥ 0 for all t, Eq 8 can be re-written as:

$$\begin{array}{l} R_{\left|EMG\right|,s}(\tau )=E\{\left|\int_{-\infty }^{\infty }p(\alpha )\cdot s(t-\alpha )\cdot s(t+\tau )d\alpha \right|\}\\ \leq E\{\int_{-\infty }^{\infty }\left|p(\alpha )\right|\cdot s(t-\alpha )\cdot s(t+\tau )d\alpha \}\\ =\int_{-\infty }^{\infty }\left|p(\alpha )\right|\cdot R_{ss}(\tau +\alpha )d\alpha \end{array}$$ (9)

Comparing Eqs 5 and 9, we get:

$$R_{\left|EMG\right|,s}(\tau )\leq R_{EMG_{nc},s}(\tau ),\forall \tau$$ (10)

Eq 10 shows that the correlation between the rectified EMG and the neural drive is always equal to or smaller than the correlation between the no-cancellation EMG and the neural drive. The two correlation levels will the same only if the action potential waveform is only positive, which would correspond to the absence of cancellation. Therefore, although the no-cancellation EMG is not a perfect estimate of the neural drive, it would provide better estimates of the neural drive to muscle than the rectified EMG. The difference between the rectified EMG and the no-cancellation EMG is the cancellation signal term (Eq 3) and cancellation determines the decrease in association between the corresponding EMG signal and the neural drive. The above derivations show that in all conditions, cancellation is detrimental to the association between EMG and the neural drive to muscle.

### Simulations

Using the computational model (Fig 1), the activity of the motor neuron population, the force, and the EMG (“regular” rectified EMG and EMG_nc_) were simulated. Based on a synaptic input signal, the cumulative spike train (CST; algebraic sum of all motor unit spike trains) representing the neural drive to the muscle [2,7], the muscle force, the rectified and the no-cancellation EMG signals were simulated. The no-cancellation EMG was obtained by summing the rectified motor unit action potentials. EMG_nc_, which cannot be experimentally derived, was used to assess the effect of amplitude cancellation on the rectified EMG when inferring frequency components of the neural drive to muscle [17]. The synaptic input to each motor neuron consisted of a signal common to all motor units and an independent noise source [2]. The common synaptic input was modeled as the sum of a series of 30 sine waves (frequency: 1, 2, 3, … 30 Hz) with random phases and amplitudes and with an offset. Across the simulations, we varied the number of motor units and the characteristics of the common input. Each combination of these settings was repeated 15 times. The amplitude of each imposed sine wave was assigned a random value in each repetition. Using linear regression analysis, the strength of the association between the power of the neural drive and each of the EMG signals (rectified EMG and EMG_nc_) at each imposed frequency was obtained across all simulated conditions.

**Fig 2:**
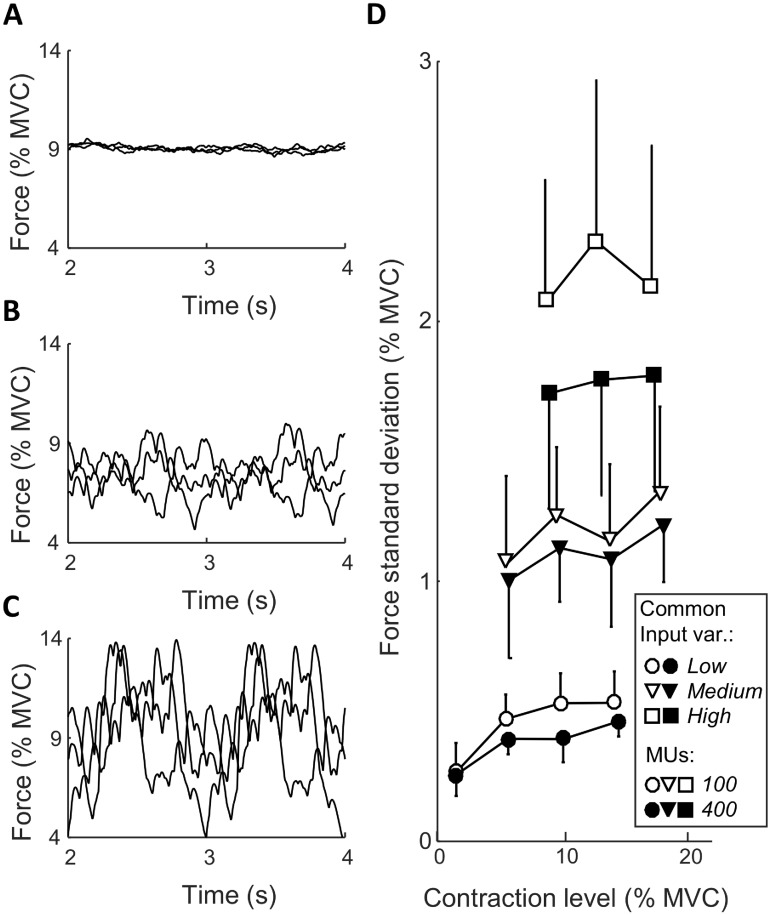
Three representative examples of simulated forces with low (A), medium (B), and high (C) amplitudes of the variability of the common synaptic input to a population of 400 motor neurons. Panel D illustrates the force standard deviation (mean±std) for all simulated conditions. Here, the symbols along each line represent different offsets of the common synaptic input and the different lines represent different amplitudes of common input variability and/or different number of motor units.

The number of recruited motor units and their average discharge rate increased with the offset of the common input. Across all simulations with the low offset (contraction level: 1.4% MVC) and low common input variability, 18% of the motor units were recruited with an average discharge rate of 5.9 pulses per second (pps). The number of recruited units and their discharge rates increased steadily as the offset increased, reaching 53% recruited motor units, on average discharging at 27.7 pps at the highest offset (contraction level: 17.1% MVC). The discharge rates across the motor unit pool were highest for the smallest motor units. An increase in the variability of the common synaptic input also determined an increase in the number of recruited motor units and their discharge rates. On average, the number of recruited motor units increased by 22 percentage points and the average discharge rate by 3.7 pps when increasing the variability of synaptic input in the analyzed range. The impact of these different recruitment patterns on the evoked force is illustrated in Fig 2. As expected, the main determinant of force variability was the amplitude of the variability of the common input to the motor neuron population. In addition, the force was less variable when simulated with a larger motor neuron population, as previously shown [20]. In the simulations with low amplitude of common input, the standard deviation of force was 0.3–0.5% of MVC, which corresponds to the variability experimentally observed when young healthy subjects are asked to maintain a steady contraction level with visual feedback [21–24]. For example, the coefficient of variation (standard deviation divided by the mean) of force is usually >5% for low contraction levels (<5% MVC) and stabilizes at <3% at higher contraction levels [21–24], which is similar to the values for low common input in Fig 2(D). Conversely, force fluctuations are expected to be substantially greater during most functional tasks, such as gait, where force modulation is needed. In this way, the simulations with higher variability of the common input (Fig 2B and 2C) represents such conditions in a more accurate way.

**Fig 3:**
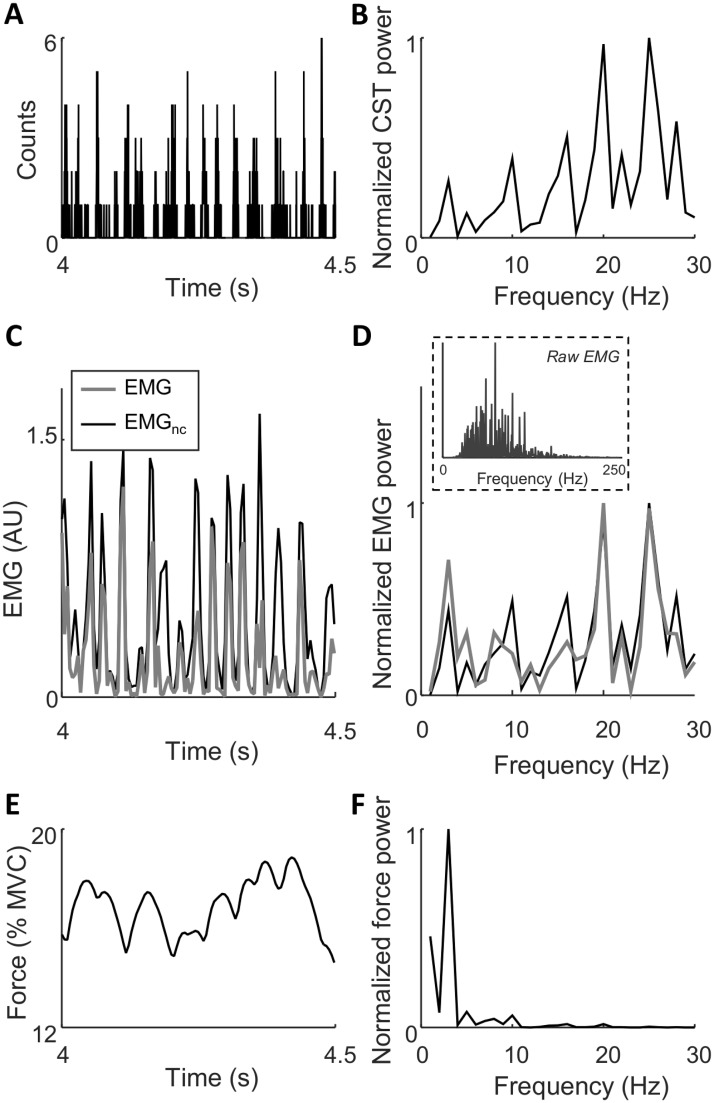
Temporal and spectral representation of the cumulative spike train (CST; A, B), the rectified EMG and EMGnc (C, D), and force (E, F). In panel D, the normalized power spectrum of the raw (unrectified) EMG signal in the 0–250 Hz range is included in the dashed box. In this example, the muscle consisted of 100 motor units, the amplitude of the variability of the common synaptic input was high (force standard deviation: 1.8%MVC), and the average contraction level was 14.9%MVC. The power spectra (B, D, F) indicate power only for integer frequencies that were the frequency components used to simulate the common input.

Fig 3 shows the neural drive to the muscle (CST), the EMG signals and the force in the time and frequency domain in one simulation. The median frequency of the raw EMG signal over all simulations with low common input variability was 72.0±14.8 Hz, which is within the range of experimentally observed values [25–27]. This figure enables a visual comparison between the characteristics of the signals, and the degree to which the neural drive is reflected in the other signals. The variation in the power of the neural drive across the different frequencies reflects the random amplitudes of the sine waves composing the common synaptic input [2]. In the time-domain, the amplitude of EMG_nc_ was always greater than for the rectified EMG indicating the effect of amplitude cancellation (Fig 3C). The two EMG signals, however, were relatively similar and usually exhibited peaks at the same time instants. Consequently, their spectral characteristics were similar, but not identical (Fig 3D). The power spectrum of the EMG_nc_ closely resembled that of the neural drive (Fig 3(B)), to a greater extent than for the rectified EMG. This illustrates the distortion imposed by amplitude cancellation in the frequency domain. As expected, the low-pass filtering properties of the motor unit twitches implied that only the lowest frequency components of the neural drive were transmitted to force (Fig 3F).

**Fig 4:**
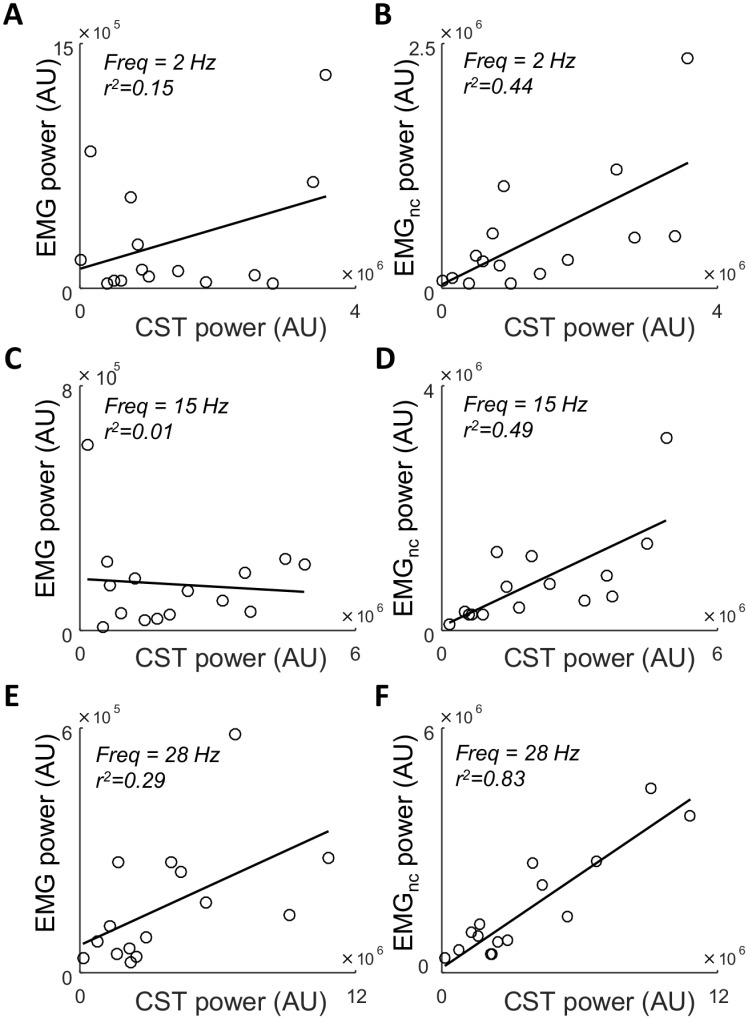
Linear relations between the power at three frequencies (2 Hz: A, B. 15 Hz: C, D. 28 Hz: E, F) in the CST and the rectified EMG (A, C, F) as well as the CST and EMG_nc_ (B, D, F). The data in these examples represent simulations in which the muscle consisted of 100 motor units, the amplitude of the variability of the common synaptic input was high (force standard deviation: 1.8%MVC), and the average contraction level was 14.9%MVC. In each panel, each circle represents the power of the two signals in one of the 15 simulations conducted with these parameter values.

**Fig 5:**
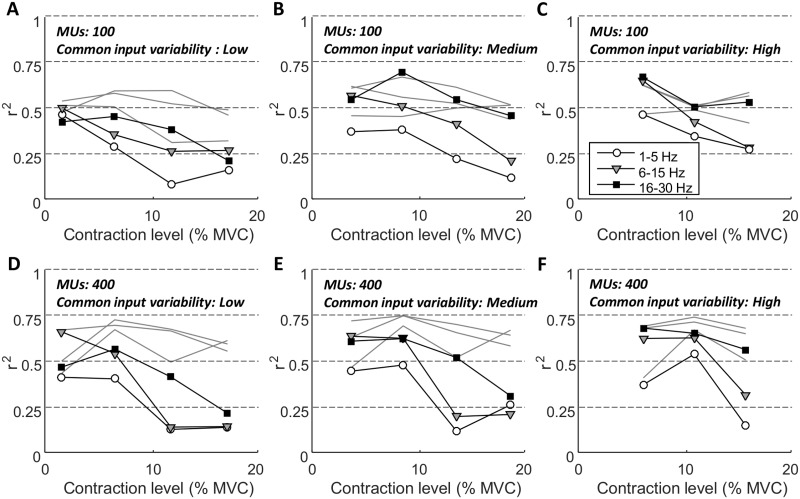
Values of r^2^ for the linear relations between CST and rectified EMG and EMG_nc_ respectively, as functions of the contraction levels across all simulations. In each panel, black lines represent the relation between CST and rectified EMG (symbols represent average values for the 1–5 Hz, 6–15 Hz, and 16–30 Hz frequency bands, respectively; see inset in panel C), while grey lines represent the relation between CST and EMG_nc_ (each line represents the same frequency bands as for CST-EMG). Panels A, B, C show results for simulations with 100 motor units, while panels D, E, F represent 400 motor units. Panels A, D represent low variability of the common synaptic input to the motor neurons, panels B, E medium variability, and panels C, F high variability.

Fig 4 shows examples of the linear relation between the distribution of power across frequencies for the CST and for the two EMG signals across 15 repetitions. Specifically, the power at 2, 15, and 28 Hz are shown for simulations with high common input amplitude. For the EMG_nc_, an association was present for these three frequencies (r^2^≥0.44), whereas the association was much weaker for the rectified EMG. For the rectified EMG, however, the linear correlation was strongest at the highest frequencies (r^2^ = 0.15 at 2 Hz, Fig 4(A); r^2^ = 0.29 at 28 Hz, Fig 4(E)). These observations are confirmed by average values of coefficient of determination across all simulations (Fig 5). Here, average r^2^ is represented as a function of contraction level for different frequency bands across the simulation conditions. Across all conditions, the correlation between EMG_nc_ and CST was high (mean r^2^ = 0.59±0.08) and was largely unaffected by contraction level. Conversely, the correlation between EMG and CST was relatively high at low contraction levels (r^2^≥0.37), and decreased greatly when the contraction level (and thus amplitude cancellation) increased. This trend was observed across all conditions, but the decrease was strongest when the amplitude of the common input was low. For example, r^2^ was 0.19±0.05 with low common input and 0.35±0.16 with high common input, for contraction levels >10% MVC. The lowest correlations were typically observed for the lowest frequencies. For example, at contraction levels between 5–10% MVC, r^2^ for the beta band (16–30 Hz) was on average 0.32±0.14 higher than for the delta band (1–5 Hz). Finally, the number of motor units did not have a large effect on the values of r^2^.

**Fig 6:**
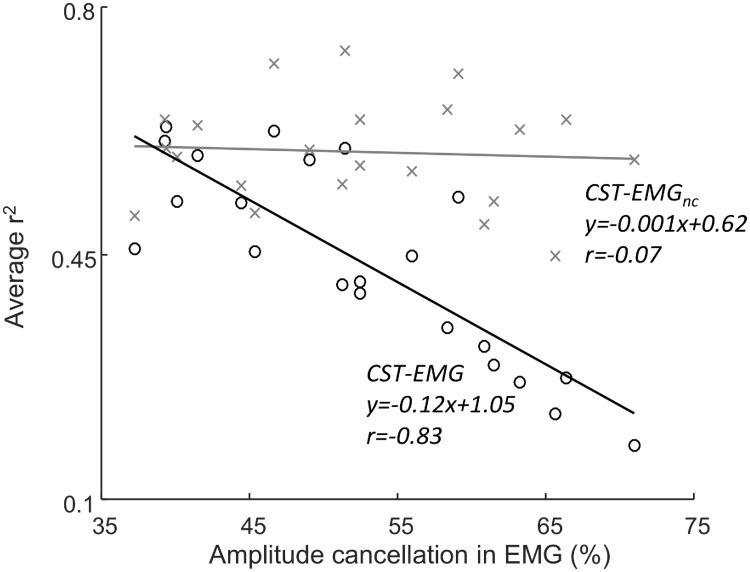
The relation between the average r^2^ (across all frequencies) for CST-EMG and the degree of amplitude cancellation in the EMG (black circles) across all simulation conditions. In addition, the average r^2^ for CST-EMG_nc_ (grey x’s) is included. Here, the value on the axis represents the level of amplitude cancellation from the EMG in the same simulation.

The values of amplitude cancellations across all simulations were between 37 and 71%, which corresponded to the ranges reported in previous simulation studies [17,18]. In general, the lowest levels of amplitude cancellation were achieved with low contraction levels and high common input. The level of amplitude cancellation was strongly negatively correlated with the average correlation between CST and rectified EMG across all imposed frequencies (r = -0.83; Fig 6). While the values of r^2^ were similar to those of EMG_nc_ (≥0.5) at low levels of amplitude cancellation (<40%), these values decreased at higher levels of amplitude cancellation. Conversely, in the simulations where the correlation between the rectified EMG and CST was low (i.e., those with high levels of amplitude cancellation) the correlation between EMG_nc_ and CST was unaffected.

## Discussion

In this study, we systematically investigated the relation between the magnitude of amplitude cancellation in the rectified surface EMG signal and the ability of this signal to reflect the neural drive to the muscle. The neural drive is the ensemble of discharge timings of all motor neurons innervating the muscle. We imposed common synaptic input to the motor neurons in the frequency band 1–30 Hz and therefore analyzed the neural drive in this frequency range. First, using an analytical approach we demonstrated that amplitude cancellation implies that the rectified EMG is not an optimal estimator of the neural drive. Next, the simulation results indicated that amplitude cancellation strongly impairs this ability to a degree where some frequency components of the neural drive would only be weakly present in the EMG (Fig 6). As also shown in previous studies, the level of amplitude cancellation was low when few motor units were active, but increased when more motor units were recruited [17,18]. This trend was clearly reflected in the simulations, where the correlation between the frequency components in the neural drive and in the rectified EMG was high at low contraction levels, but decreased rapidly when the contraction level increased (Fig 5). An important finding of the study was that the rate of this decrease depended on the strength and frequency band of the neural drive component. Specifically, this implies that strong oscillating components in the neural drive are required to overcome the distortion arising due to amplitude cancellation. Moreover, the result suggests that low-frequency components of the neural drive in the rectified EMG are more susceptible to this distortion. Finally, the simulations confirmed the observation from the derivations (Eq 7) that even without amplitude cancellation, the EMG signal does not provide a perfect characterization of the neural drive (average r^2^ for EMG_nc_ and CST was 0.59), because of the effect of the shape of the action potentials.

These finding can explain a number of previous experimental observations. For example, although the neural drive during steady isometric contractions often contains strong low-frequency components (<5 Hz) [1], these are typically not observed in coherence analyses involving the rectified EMG [28–31]. Conversely, in dynamic tasks such as gait, where strong low-frequency components of the neural drives determine substantial force fluctuations [7], high levels of EMG-EMG coherence (indicating strong shared neural drive between two EMG signals) can be observed in the delta band [32–36]. Similarly, when subjects are asked to generate a steadily increasing isometric force from rest to maximum (i.e., the strongest low-frequency variation in the neural drive that can be generated), the EMG amplitude typically displays an increasing trend similar to that of force [37–39]. Together, these observations indicate that low-frequency components of the neural drive can be detected by the rectified EMG, but only if their power is sufficiently high. Furthermore, whereas low-frequency components of the neural drive appear to be disrupted in the rectified EMG during steady contractions, neural inputs to motor neurons at higher frequency bands (>20 Hz) have been observed in such conditions from EMG analysis [15,28–31], which supports the finding that EMG amplitude cancellation primarily distorts the low frequencies.

Overall, the results identify a limitation related to the analysis of the neural drive to muscles based on the EMG signal. This limitation has implications for many methods for investigating the neural control of movement. Such applications include muscle synergy analysis that investigates modular control of activation of multiple muscles [40,41]. In such analysis, the rectified EMG signals are usually low-passed filtered—sometimes with cut-off frequencies as low as 4 Hz [42]—which implies a risk that the signals considered (only the lowest frequencies) are heavily corrupted due to amplitude cancellation. This may particularly be the case when applied to conditions with little muscle force variability such as postural control [43,44], whereas applications such as gait [45] likely involves sufficiently large variations in the low-frequency content of the neural drive to minimize the distortion of the EMG spectrum. Similarly, the results also have implications for the comparison between the rectified EMG and mechanical measures (force/acceleration), which have been used, e.g., to explain the role of neural oscillations in physiological tremor [46]. It should be underlined, however, that this study does not suggest that all analyses of rectified EMG signals are invalid. Conversely, the results imply that the rectified EMG is often exposed to some degree of distortion due to amplitude cancellation, which may affect study outcomes to varying degrees depending on the characteristics of the motor task.

Since amplitude cancellation reflects the amount of overlap of motor unit action potentials, its magnitude can be reduced by increasing the selectivity of the recording and/or recording derivation. The most selective interface is intramuscular EMG, where recordings with almost no overlap between action potentials can be achieved at low contraction levels [47]. Although the amplitude cancellation, and thus the distortion of the EMG signal, is minimal in such recordings, the high selectivity may imply a poor representation of the neural drive from a global analysis of the signal properties. Depending on the contraction characteristics, the activity of a critical number of motor units needs to be reflected in the signal for it to accurately represent the neural drive [2]. In this way, increasing the recording selectivity as a solution to this problem implies a trade-off between reducing amplitude cancellation and maintaining a sufficient number of motor units contributing to the signal. It is, however, unknown, what characterizes the optimal trade-off between these factors and if a precise characterization of the neural drive can be achieved, even in the optimal case. Instead, a more plausible solution to the problem could be to base the analysis on motor unit spike trains rather than on EMG [2]. Although the process of spike train identification adds an additional level of complexity to experimental protocols, recent advances in EMG decomposition enable non-invasive identification of large numbers of spike trains [8–10].

In the theoretical analysis, the EMG signals were analytically described on the basis on one action potential waveform (Eqs 1 and 2). This is a simplification with respect to experimental conditions where action potentials across a motor unit pool exhibit variations in amplitude as well as duration [48]. Whereas such variations will affect the filtering properties of the action potentials (Eq 7), they will not impact on the main conclusion drawn from the theoretical analysis. Indeed, the theoretical predictions have been fully confirmed by the numerical simulation results, that were achieved using realistic action potentials for each individual motor unit.

The primary limitation of the model is that the simulated discharge rate of the motor units does not saturate above a critical excitation limit. Experimentally, this is observed when discharge rates of motor units recruited at low forces cease to increase with increasing force, while the discharge rate of motor units recruited at higher forces continue to increase [49]. Conversely, in the model, the discharge rates of all motor units (once recruited) increase monotonically with input excitation level. The rate at which motor unit discharges saturate varies substantially across muscles [50], but the simulated discharge rates for the smallest motor units at the highest contraction levels (approximately 30 pps) are above the rates that would normally be assumed as their maximum [24]. In spite of the fact that the discharge rates of low-threshold motor units at some point cease to increase, evidence suggests that their discharge rates continue to reflect variation in the common synaptic input [51]. To the best of our knowledge, no computational motor neuron model accurately captures this behavior. Instead, we selected the model with the most realistic amplitude and phase response [52] and limited the simulations to relatively low contraction levels where discharge rates of low-threshold motor units did not greatly exceed those observed experimentally. Nevertheless, the failure of the model to fully recreate realistic conditions may have biased the results. When the simulated discharge rates of low-threshold motor units exceeded the rate required for tetanic force (approximately 20–24 pps [53]), an increase in the offset of the synaptic input would imply not only recruitment of new motor units (serving to increase the contraction level) but also additional action potentials discharged by low-threshold units (not generating more force). Thereby, the density of action potentials, and thus the level of amplitude cancellation, would be higher than in experimental conditions for that particular contraction level. This would imply that the simulations to some degree overestimated the rate by which the correlation between the rectified EMG and the CST declined with contraction level (Fig 5). However, we believe that the impact of this error on the outcome of the study is relatively small. Indeed, first, in most simulation conditions the correlation already began to decline at approximately 12% MVC where simulated discharge rates presumably corresponded to natural conditions (Fig 5). Second, the additional action potentials at the highest simulated contraction levels are those with the lowest amplitude due to the relatively low innervation numbers of low-threshold motor units. Therefore, their potential impact on the EMG signal is minimal.

Finally, a limitation of the simulation strategy is that, the average amplitude of the sine waves imposed as common synaptic input to all motor neurons across the 15 repetitions was uniform for all frequencies. This assumption may not apply to experimental conditions. Since synaptic inputs at different frequency bands are often attributed to specific neural structures [51,54,55], some frequency-dependent variability in the amplitude would be expected across tasks. For example, synaptic input at approximately 8 Hz has been attributed to stretch-sensitive afferent feedback [56], whose synaptic strength can be modulated by presynaptic inhibition in task-dependent ways [57]. In addition, the strength of the synaptic input in the beta band also depends on the motor task [58]. This implies that the relation between the variability of the neural drive and the muscle force (Fig 2) cannot be generalized to all conditions. For example, it is possible that a contraction with low variability of synaptic input at low frequencies (i.e. little force variability) has a strong beta band input. In this case, the force steadiness cannot be used as a measure for the potential distortion of the spectral components of the rectified EMG at high frequencies. However, the linearity of the motor neuron pool [2–4] implies that the components of the neural drive reflecting synaptic input from different neural structures can be identified more accurately from the CST than from EMG.

In conclusion, the study demonstrates that amplitude cancellation impairs the degree to which the rectified EMG signal reflects the neural drive to the muscle, except in conditions with low contraction levels and/or highly variable common synaptic input. This distortion of the EMG signal affects primarily its lowest frequency components.

## Methods

### Computational model

The model architecture is illustrated in Fig 1. To summarize, the synaptic input to the pool of motor neurons determined their discharge pattern. Based on those patterns, the force, EMG, and EMG_nc_ were simulated.

### Motor neuron model

The number of motor neurons was set to 100 or 400. Each motor neuron was simulated with Hodgkin Huxley-type models [59,60] and consisted of two compartments (soma and dendrite) for motor neurons with six conductances (leak conductances for the soma and dendrite, compartment coupling conductances between the 2 compartments, and 3 voltage-dependent conductances, sodium Na, fast potassium Kf, and slow potassium Ks) and four state variables (m, h, n, q). Membrane-specific capacitance was set to μ1 F and axial resistivity to 70 Ωcm. The soma-specific resistance ranged from 1.15 to 0.65 kΩcm^2^ and the dendrite-specific resistance from 14.4 to 6.05 kΩcm^2^. Equilibrium potentials were 120 mV for sodium and 10 mV for potassium, while the equilibrium potential of the membrane and leakage voltages were 0 mV. Input to the motor neurons was simulated as injected currents into the soma compartment. The ranges of model parameters were adopted from a previous study [61] with exponential distributions across the motor neuron population (i.e. many low-threshold motor units, few high-threshold motor units) [53]. The differential equations of the model were solved in MATLAB 2015a with the function “ode15s.”

The simulated motor neuron spike trains were used as inputs to simulate EMG and force. In addition, the algebraic sum of all motor unit spike trains (CST) was obtained as a representation of the neural drive to the muscle.

### EMG model

A library of motor unit action potentials was simulated using a previously proposed model [62]. The model included 101,276 individual muscle fibers (average length: 100 mm) with innervation zones distributed in a 10 mm region around half of the fiber length. The muscle fibers were located in a cylindrical shape (thickness: 27.5 mm; conductivity: 0.1 S/m in radial and transverse directions, 0.5 S/m in longitudinal direction) around a bone (radius: 7.5 mm; conductivity: 0.02 S/m). In addition, a subcutaneous layer (thickness: 1 mm; conductivity: 0.05 S/m) and skin (thickness: 2 mm; conductivity: 1 S/m) surrounded the muscle. For the simulations, 15 electrode pairs (circular; radius: 2 mm; inter-electrode distance: 10 mm) were included equally distributed around this cylinder halfway between the innervation zone and the end of the fibers. For each electrode, 100 or 400 motor units were selected within a circular area (radius: 7 mm or 14 mm, respectively) centered 5.3 or 10.5 mm below each electrode. For each motor unit pool, the innervation numbers were exponentially distributed (range: 6–69). In this way, 15 different sets of motor unit action potentials were generated for each of the two muscles consisting of 100 or 400 motor units. Interference EMG signals were simulated as the sum of the motor unit action potential trains (convolution of the motor neuron spike trains with the action potentials assigned to that motor neuron). In addition to the interference EMG signal, a EMG_nc_ was simulated by rectifying the action potentials prior to convolution with the spike trains.

### Force model

Muscle force was simulated based on the motor unit spike trains using a previously proposed model [53]. According to this model, the motor unit twitches were modelled as critically damped second-order system where the peak amplitude varied 100-fold across the motor unit population. The contraction time (time from twitch onset to peak) ranged from 30 to 90 ms. Both twitch parameters were distributed according to an exponential relation, so there were most low-amplitude, slow-twitch units. In addition, the gain of each twitch depended in a non-linear way on the instantaneous motor unit discharge rate.

The MVC force was estimated in pilot simulations as the force elicited by the muscle when all motor units were active at a discharge rate equivalent to the peak discharge rate proposed in previous simulation studies [53].

### Simulation strategy

The synaptic input to each motor neuron consisted of a common and an independent term and an offset. The independent term was low-passed filtered (<100 Hz) white Gaussian noise scaled in order to get a realistic variability of the inter-spike intervals (coefficient of variation: 10–30% [22,63,64]) in the absence of common input. The common synaptic input was the sum of 30 sine waves (frequency: 1–30 Hz) with random phases. This range of frequencies were selected as they represent those most often analyzed in experimental conditions. The gains G1_F1-F30_ (Fig 1) were random values selected from a uniform distribution between 0 and 1. The gain G2 determined the average variability of the common input and was scaled to get different values of the strength of the common input and of the variability of force. Across simulations, three different values were assigned to G2: 5.7·10^−5^, 1.5·10^−4^, 2.4·10^−4^. These values ensured standard deviations of force equivalent to those observed when subjects aim to maintain stable force with visual feedback and equivalent to more functional, force-varying tasks, respectively (see *Results*). The offset was scaled to simulate different average contraction levels and was assigned the values 3.5·10^−3^, 3.8·10^−3^, 4.0·10^−3^, 4.3·10^−3^. These values ensured average contraction levels between 1 and 20% MVC. All combinations of these values for G2 and offset were simulated using motor neuron populations consisting of 100 and 400 motor units, respectively. The combination with the highest G2 (2.4·10^−4^) and the highest offset (4.3·10^−3^) was excluded since it evoked contraction levels >20% MVC and thereby involved unrealistic motor unit discharge patterns (see *Discussion* for details). In total, this implied 22 simulated conditions. Each condition was repeated 15 times. In each of these repetitions, the independent synaptic noise, the phase of each sine wave, and the values assigned to G1_F1-F30_ varied. Furthermore, the EMG signals for each of these repetitions were based on different sets of motor unit action potentials (see *EMG model* for details).

### Analysis

Across the 15 repetition of each simulation condition the amplitude of each of the 30 imposed frequencies (1–30 Hz) were extracted from the power spectra of the CST and the two EMG signals (rectified EMG and EMG_nc_). The linear correlations between these power amplitudes of the CST and the rectified EMG, and the CST and EMG_nc_ were analyzed separately for each frequency. For each simulation condition, the average values for r^2^ in the 1–5 Hz band (delta band), the 6–15 Hz band (alpha band), and the 16–30 Hz band (beta band) were calculated. In this way, the association between the neural drive and the EMG was identified across input frequencies. For each condition, the level of amplitude cancellation was calculated as the ratio between the average values of the cancellation term (Eq 3) and EMG_nc_.
